# Electrochemical Migration Behavior of Ag Electrodes in Negative Temperature Coefficient (NTC) Sensors Under Complex Environmental Conditions

**DOI:** 10.3390/ma19214510

**Published:** 2026-10-26

**Authors:** Bin Liu, Tianjun Wang, Zhuo Peng, Ye Du, Chunyan Huang, Fei Wu, Feifei Dong, Bo Li

**Affiliations:** 1 Tsinghua Shenzhen International Graduate School, Shenzhen 518055, China; 2 Shenzhen Hoverbird Electronic Technology Co., Ltd., Shenzhen 518110, China

**Keywords:** electrochemical migration, Ag electrode, NTC sensor, Ag dendrites

## Abstract

To investigate electrochemical migration (ECM) and related failure of Ag electrodes in negative temperature coefficient (NTC) sensors under complex environments, screen-printed coplanar Ag electrodes were tested by means of real-time resistance monitoring and in situ CCD (Charge-Coupled Device) observation. The effects of water films, applied bias, NaCl concentration, and solution pH were examined. No observable migration occurred at 80 °C and 3 V without liquid water. In pure water, Ag dendrites grew from the cathode toward the anode and formed conductive bridges; increasing the bias from 3 to 4 V shortened the time to short circuit. In neutral NaCl media, 0.1 mmol L^−1^ NaCl allowed Ag-containing species to migrate across the electrode gap and form dendrites, whereas 1 and 5 mmol L^−1^ NaCl promoted AgCl-related deposits that suppressed sustained Ag dissolution, with the extent of inhibition depending on dissolution flux and surface coverage. In HCl media, surface deposits provided insufficient long-term protection, leading to continued Ag dissolution and short-circuit failure. In Na_2_CO_3_ media at pH = 9 and pH = 12, insoluble Ag-containing deposits formed on the anode and no dendritic bridging was observed. Overall, Ag ECM is governed by the competition among anodic dissolution, surface deposition/passivation, and the transport of Ag-containing species across the electrode gap. The results provide a basis for material selection, package design, and reliability assessment of NTC sensors for power batteries.

## 1. Introduction

NTC thermistor sensors are important temperature-monitoring components in battery thermal management systems, and their long-term reliability directly affects thermal safety management of battery systems [1]. Under service conditions involving elevated temperature and humidity, electrical bias, and contaminant ingress, Ag electrodes in such sensors may undergo electrochemical migration (ECM) [2,3,4]. In this process, anodic Ag is oxidatively dissolved in the presence of an electric field and a liquid or adsorbed water film, producing mobile silver-containing ions or complexes. These species are subsequently reduced and deposited near the cathode, progressively forming conductive dendrites or metallic bridges, which eventually lead to decreased insulation resistance, leakage current, and even short-circuit failure [2,5]. Therefore, elucidating the migration behavior of Ag electrodes and its governing mechanisms under complex environmental conditions is important for improving the service reliability of NTC sensors.

Actual service environments are highly diverse and dynamic. For example, salt spray or saline condensate may form Cl^−^-containing thin liquid films on device surfaces [6]; certain encapsulants, adhesives, or adjacent materials may release volatile organic acids during thermal aging, thereby altering the local acid–base environment; and coolant leakage, cleaning residues, or alkaline contaminants may increase the local pH. These chemical environments not only modify the conductivity of the liquid film, but also substantially affect the likelihood, rate, and morphology of ECM by influencing anodic Ag dissolution, complexation, precipitation, and cathodic reduction [7,8]. In Cl^−^-containing environments, low Cl^−^ concentrations may promote migration by increasing ionic conductivity or forming mobile silver–chloride complexes, whereas AgCl deposits formed at higher Cl^−^ concentrations may impede anodic dissolution [9]. This transition from promotion to inhibition is not universal; its critical conditions are also affected by bias voltage, humidity, liquid-film thickness, oxygen availability, and electrode configuration. Likewise, the effect of acid–base conditions on Ag migration cannot be assessed solely based on pH, but should be considered in conjunction with the stability of Ag species, precipitation reactions, and interfacial electrochemical kinetics.

Considerable efforts have been devoted to Ag ECM. Kohman et al. [5] reported the migration of metals under the combined action of moisture and an electric field, identifying the water film as a prerequisite for ionic transport and electrochemical reactions. However, the role of Cl^−^ remains under debate. Some studies have suggested that dissolved salts increase the conductivity of the liquid film and decrease ohmic resistance, thereby facilitating ionic transport and dendritic growth. Others have shown that, within certain concentration ranges, sparingly soluble products such as AgCl may form on the anode surface, restricting sustained Ag dissolution and delaying short-circuit failure [10]. Using a water-drop method, Xu et al. [11] investigated the ECM behavior of sintered nano-Ag layers and observed a gradual transition in migration-product morphology from feather-like to pine-needle-like structures with increasing Cl^−^ concentration; AgCl coverage at the anode was also found to possibly retard Ag dissolution. Luo et al. [12] examined migration in a Cu/Ag system under NaCl thin-film conditions and reported that the short-circuit time increased with NaCl concentration but decreased with applied voltage. These findings indicate that ECM is not governed by a single environmental variable, but rather by the coupled competition among anodic metal dissolution, precipitation of reaction products, ionic migration and diffusion, and cathodic reduction and deposition. Nevertheless, existing studies have largely focused on individual contaminants or specific metallic systems. Systematic investigations of Ag electrodes in NTC sensors under the combined effects of temperature–humidity conditions, electrical bias, salts, and acid–base contaminants remain limited. In particular, a unified framework is still needed to explain how environmental factors alter anodic interfacial reactions, the concentration of mobile Ag species, and the conditions for dendrite growth.

Accordingly, this study investigates the ECM behavior of Ag electrodes under typical service conditions relevant to NTC sensors, including different humidity levels, applied voltages, NaCl concentrations, and acid–base environments. By controlling the environmental temperature and humidity, bias voltage, NaCl mass fraction, and solution pH, the failure characteristics of Ag electrodes were examined under elevated temperature and humidity, biases of 3 and 4 V, NaCl concentrations of 0.1–5 mmol/L, acidic conditions of pH 2–5, and alkaline conditions of pH 9–12. Screen-printed Ag electrode substrates were employed, and the initiation, evolution, and short-circuiting processes of ECM were characterized by means of CCD observation and real-time insulation-resistance monitoring. Thermal treatment and hydrochloric acid dissolution tests were further used to assess the composition and stability of migration products. On this basis, a competitive framework was developed by considering the driving force for anodic dissolution, the capture of Ag species by precipitated products, and the diffusive transport capability of mobile species. A reaction–diffusion characteristic length was further introduced to provide a semi-quantitative assessment of migration tendencies under different environments. The results provide a reference for migration-resistant design, reliability evaluation, and failure analysis of Ag electrodes in NTC sensors.

## 2. Experimental Methods

### 2.1. Materials and Sample Preparation

Alumina ceramic substrates with a purity of 99% and dimensions of 10 × 10 × 0.6 mm^3^ were used. To minimize the influence of adsorbed contaminants and residual organic impurities, the substrates were pre-fired at 1200 °C. After cooling to room temperature, the substrate surfaces were wiped with anhydrous ethanol, labeled, and stored for subsequent use.

**Figure 1:**
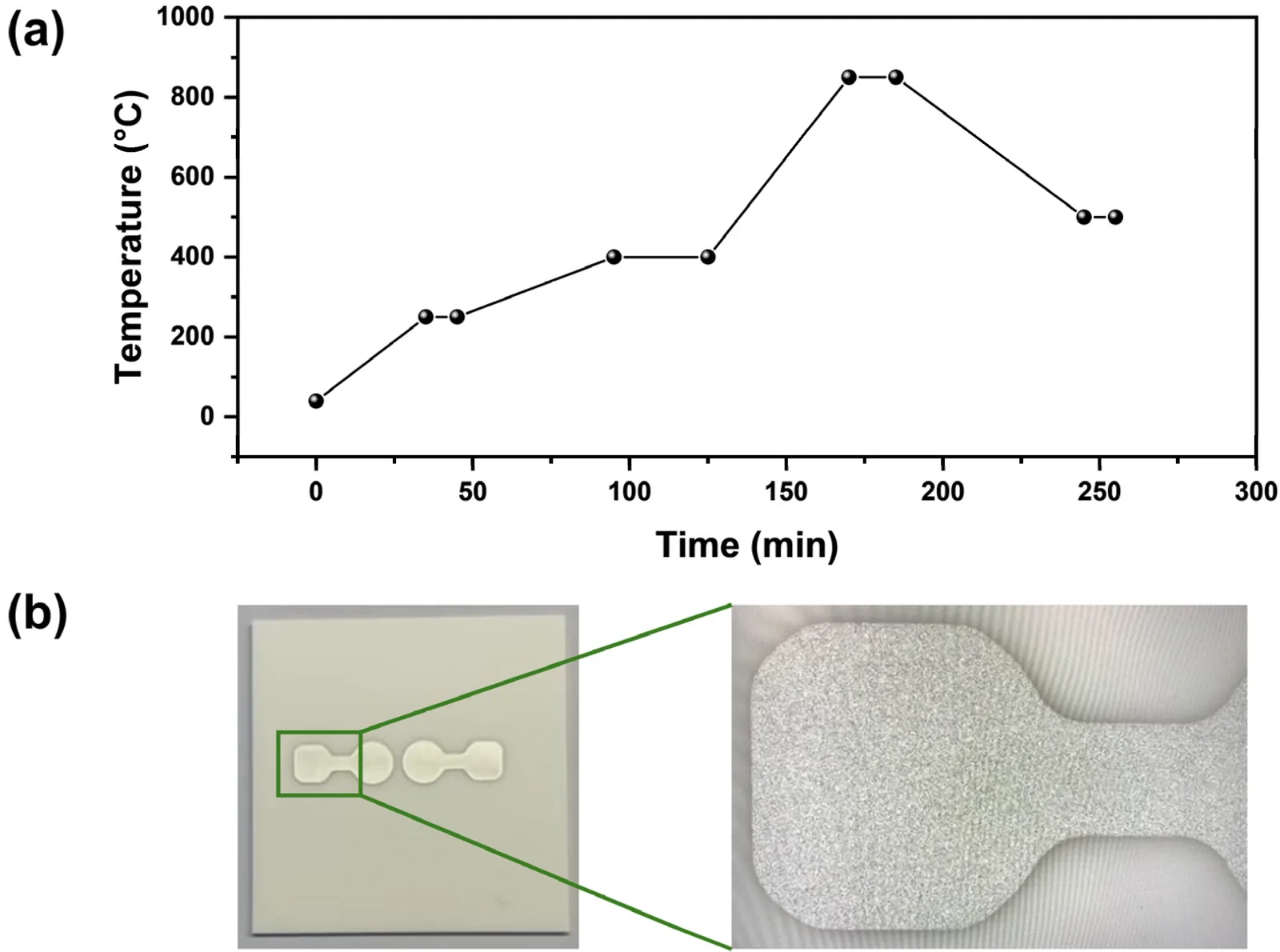
(**a**) Sintering curve and (**b**) a photograph of the Ag electrode sample.

Ag electrodes were prepared by screen printing. A commercial Ag paste (ST-007, Fenghua Advanced Technology Co., Ltd., Zhaoqing, China) designed for MLCC termination electrodes was used. During printing, the Ag paste was placed on the screen-printing stencil and transferred onto the alumina substrate using a squeegee to form coplanar Ag electrodes with a predefined pattern. The printed samples were dried at 120 °C for 60 min to remove volatile organic constituents from the paste. They were then fired in a high-temperature furnace at 850 °C for 15 min to obtain sintered Ag electrodes. The sintering curve and a photograph of the Ag electrode sample are shown in Figure 1.

A total of 11 sample groups were prepared. The electrode spacing was 1.0 mm for most samples, while selected control samples had a spacing of 2.0 mm. To assess fabrication consistency, the masses of the substrates were recorded before printing, after printing, after drying, and after firing. The mass deviation among samples was controlled within 0.1%. After firing, the electrode surfaces were examined using a CCD microscope (Kunshan Gaopin Precision Instrument Co., Ltd., Kunshan, China, GP-300C). The Ag layers were confirmed to be continuous and uniform, with no apparent pores, cracks, or unintended bridging defects. The initial insulation resistance between the two electrodes was also measured to ensure that all specimens were electrically nonconductive before testing.

### 2.2. Experimental Groups and Test Conditions

A controlled-variable approach was adopted to investigate the effects of applied voltage, NaCl concentration, and solution pH on the ECM behavior of Ag electrodes. The test conditions are summarized in Table 1. To maintain consistent liquid-layer conditions, all samples were placed on the same horizontal surface. A graduated dropper was used to apply 0.05 mL of solution at the midpoint between the two electrodes. The wetted area was approximately 20–25 mm^2^, corresponding to an estimated liquid-layer thickness of 2–3 mm. Except for the high-temperature bias tests, all experiments were performed at room temperature, with temperature fluctuations controlled within ±1 °C. The voltage-dependent tests were conducted in a thermostatic water bath at 80 °C to simulate elevated-temperature service conditions that may occur in battery packs.

For each test, samples with soldered Cu leads were placed in the designated constant-temperature test region, and a constant DC bias was applied across the electrodes. The resistance between the electrodes was continuously recorded using a precision resistance meter (Good Will Instrument Co., Ltd., Suzhou, China, GOM 804 SMS) at a sampling interval of 1 s. Meanwhile, the interelectrode region was monitored in situ using a CCD microscope to record dendrite nucleation, growth, and short-circuit formation.

Alkaline environments were prepared using Na_2_CO_3_ solutions. Since carbonate solutions may undergo equilibrium changes through gas exchange with atmospheric CO_2_, freshly prepared solutions were used as soon as possible. During testing, the solution surface was covered with a film to reduce gas–liquid exchange, and the initial pH was measured using a calibrated pH meter.

### 2.3. Characterization of Migration Products

The black products observed on the electrode surfaces after ECM tests were subjected to preliminary qualitative analysis by thermal treatment and hydrochloric acid immersion.

First, the reacted samples were placed in a rapid-firing furnace and heated at 5 °C/min to 200 and 330 °C, respectively. Changes in the color, morphology, and apparent stability of black products were recorded. Subsequently, the reacted samples were immersed in 0.107 mol/L HCl solution for 3.5 h. Changes in surface appearance before and after immersion were documented, and the solution pH was measured before and after immersion.

## 3. Results and Discussion

### 3.1. Effects of Humidity and Applied Voltage on the ECM of Ag

The control sample (substrate No. 11) was tested at 80 °C under a 3 V bias for approximately 120 min without the addition of a test solution. During the test, the resistance between the two electrodes remained at approximately 203 kΩ. No apparent color change, deposit formation, or dendritic growth was observed by CCD microscopy. These results indicate that, under the present conditions, elevated temperature and electrical bias alone were insufficient to induce observable ECM of Ag. A continuous liquid water film, or an adsorbed water layer with sufficient ionic conductivity, is required to sustain anodic dissolution, ionic transport, and cathodic deposition.

Water plays two main roles in the ECM of Ag. First, the water film provides a transport pathway for metal ions and other charged species. Second, it participates in cathodic reactions, such as oxygen reduction or hydrogen evolution, and affects the local pH and the chemical forms of Ag species at the interface. The anodic process of Ag can initially be expressed as:

Ag → Ag^+^ + e^−^ (1)

Under locally neutral or alkaline conditions, the generated Ag^+^ may further undergo hydrolysis or reactions associated with oxide or hydroxide formation, yielding sparingly soluble Ag-containing products. It should be noted that directly describing 2Ag + H_2_O → Ag_2_O + 2H^+^ + 2e^−^ as the anodic reaction in a neutral environment is not sufficiently rigorous. The formation of Ag_2_O generally involves Ag^+^, OH^−^, and hydrolysis or precipitation processes, and the reaction pathway is affected by local pH, dissolved oxygen, applied bias, and liquid-film composition. On the cathodic side, dissolved oxygen reduction may occur:

O_2_ + 2H_2_O + 4e^−^ → 4OH^−^ (2)

When the oxygen supply is limited or the cathodic overpotential is sufficiently high, water reduction may also occur:

2H_2_O + 2e^−^ → H_2_ + 2OH^−^ (3)

The accumulation of OH^−^ near the cathode may therefore promote the formation of Ag-containing oxides, hydroxides, or related products.

**Figure 2:**
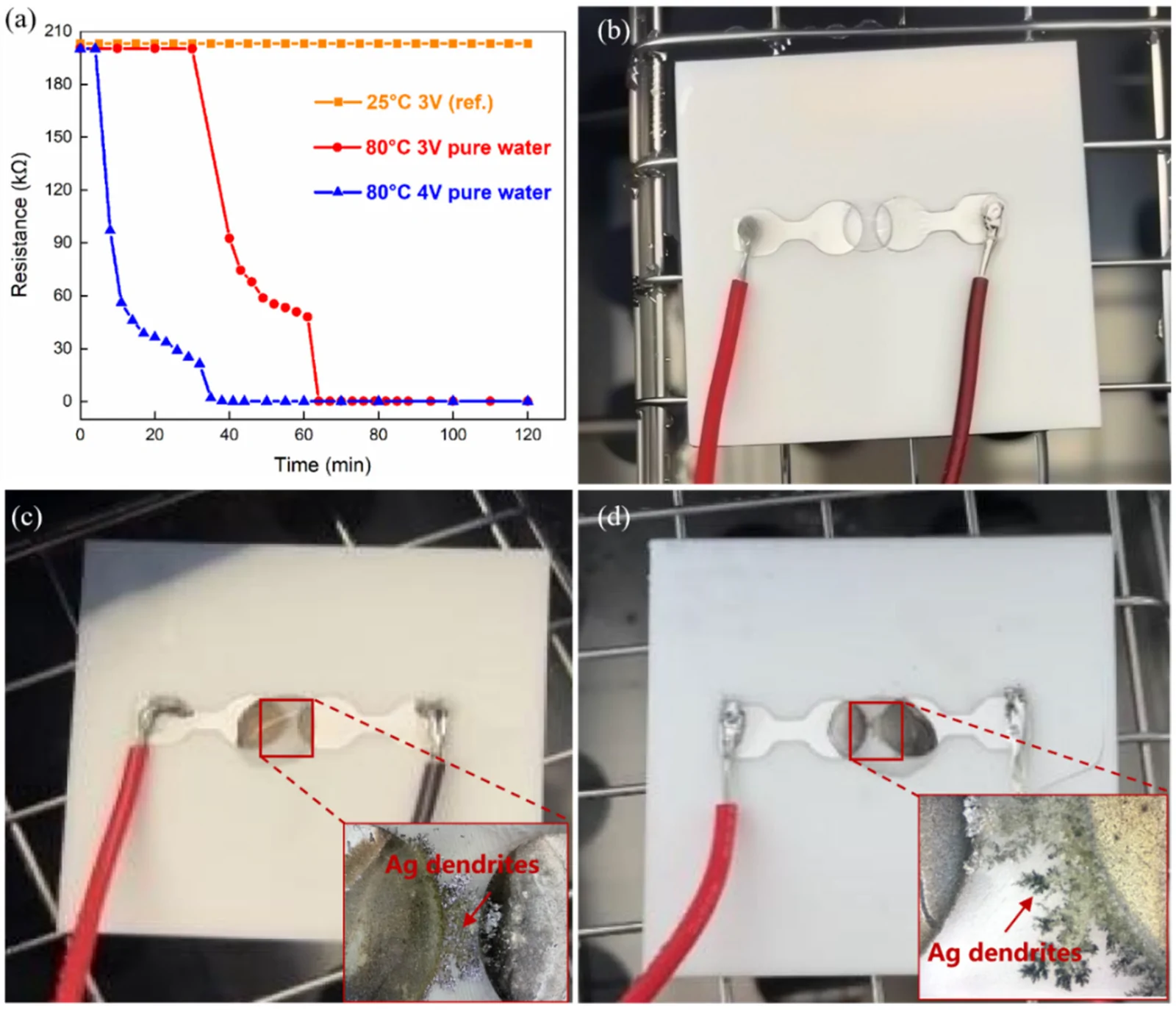
ECM behavior of Ag electrodes under high-humidity, elevated-temperature, and biased conditions: (**a**) interelectrode resistance as a function of time under different bias voltages; (**b**) CCD image of substrate No. 11 after testing at 3 V and 80 °C without addition of a liquid drop, showing no dendrite formation; (**c**) CCD image of substrate No. 1 after testing with a pure-water drop at 3 V and 80 °C, showing dendritic deposits; (**d**) CCD image of substrate No. 2 after testing with a pure-water drop at 4 V and 80 °C, showing dendritic deposits.

At 80 °C, substrate No. 1 exhibited pronounced ECM after a drop of pure water was applied and a 3 V bias was imposed. Black products appeared near the anode after approximately 10 min. The interelectrode resistance remained above 140 kΩ during the first 40 min, then decreased and reached approximately 0.1 kΩ at about 64 min (Figure 2a). This abrupt decrease indicates the formation of a continuous conductive path between the electrodes. CCD images further showed dendritic deposits extending from the cathode toward the anode, indicating that Ag-containing species generated at the anode were transported under the electric field and subsequently reduced and deposited near the cathode, eventually forming a bridging structure. When the bias voltage was increased from 3 to 4 V, the short-circuit time was reduced by approximately 20 min (Figure 2b). As shown in Figure 2c,d, distinct dendritic deposits and black surface products were observed under both voltage conditions. Within the investigated range, increasing the applied voltage clearly accelerated the ECM of Ag.

The conductivity of nominally pure water may vary with temperature, dissolved atmospheric CO_2_, ions released from the electrodes, and reaction products. In addition, the current in a water-drop test is jointly governed by ohmic drop, concentration polarization, and charge-transfer processes. Therefore, the ECM behavior at 3 and 4 V should not be regarded as being controlled exclusively by ohmic polarization, nor should current density be assumed to be strictly proportional to the applied voltage. A more appropriate interpretation is that a higher bias increases the electric-field strength and interfacial overpotential, thereby enhancing anodic Ag dissolution, electromigration of ionic species, and cathodic reduction and deposition. Consequently, the time required for dendrite formation and short-circuit failure is reduced. Liu et al. [7] likewise reported that, in low-conductivity ultrathin liquid films, liquid-film resistance and its dynamic evolution during the reaction substantially affect the ECM of Ag.

An increased anodic dissolution rate raises the generation flux of mobile Ag-containing species in the liquid film and alters the concentration and electric-field distributions near the cathode. During cathodic deposition, microscopic surface protrusions usually experience locally enhanced electric fields and mass-transport fluxes, making them preferential sites for metal deposition. This geometric amplification promotes the continued growth of protrusions into dendritic structures. Although this process can be regarded as a morphological instability during electrodeposition, its initiation and development are not controlled solely by diffusion; electromigration, local space-charge effects, interfacial reaction kinetics, and liquid-film evaporation may also contribute. Studies by Kharbachi et al. [13] have shown that space-charge effects can substantially influence the morphological stability and dendritic growth modes of metal electrodeposition in weakly supported or unsupported electrolyte systems. Accordingly, a higher bias may promote rapid dendrite growth by increasing the generation and transport fluxes of Ag-containing species and intensifying local electric-field concentration, leading to earlier short-circuit failure.

**Figure 3:**
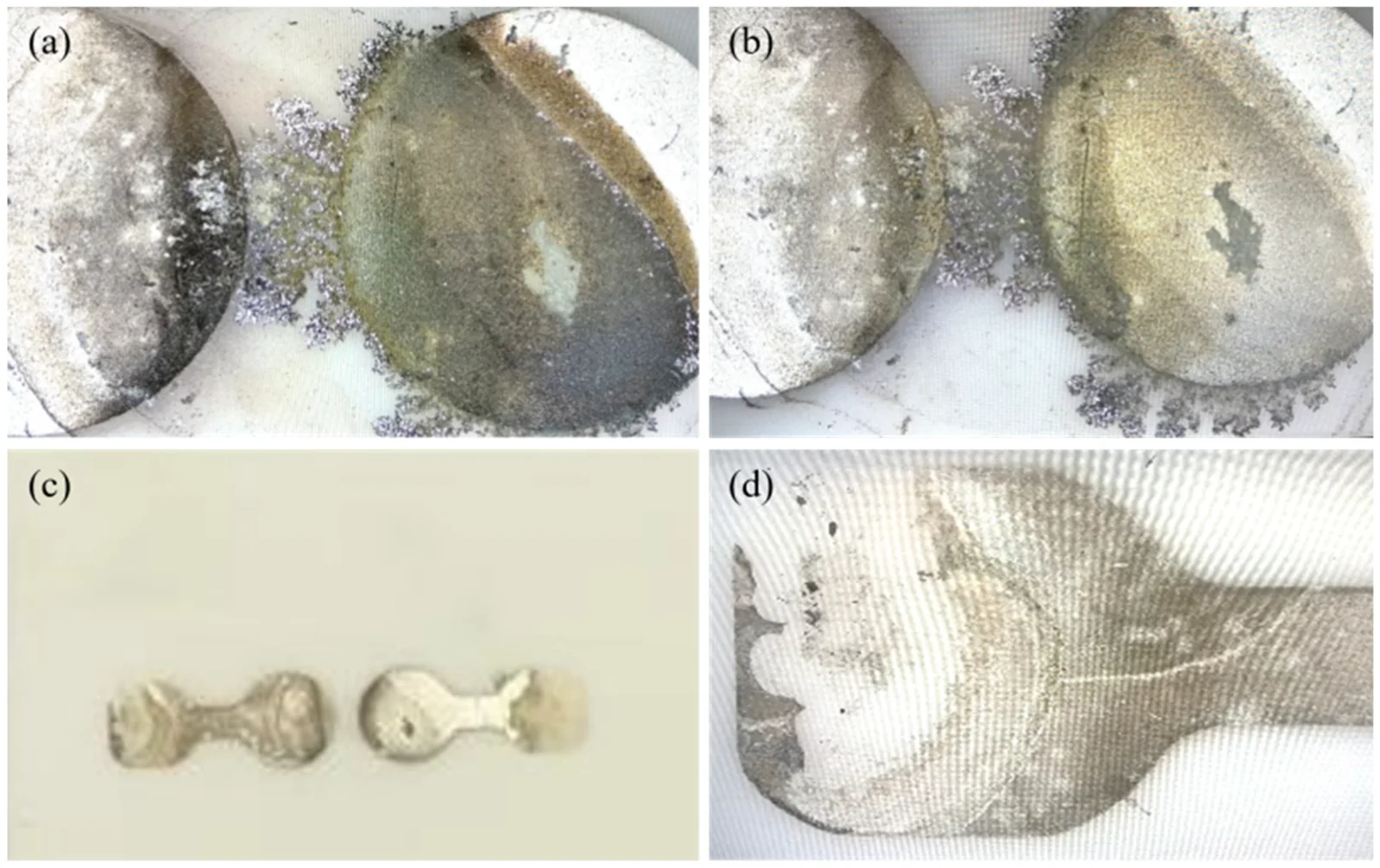
Preliminary identification of black products on the Ag electrode surface of substrate No. 2: (**a**) CCD image before thermal treatment, showing abundant black products; (**b**) morphological changes during thermal treatment at 200–330 °C, with partial lightening or disappearance of the black products; (**c**) surface morphology after thermal treatment and before HCl immersion; (**d**) surface morphology after immersion in 0.107 mol/L HCl for 3.5 h, showing a decrease in black products accompanied by the formation of white deposits.

To preliminarily assess the composition of the black products formed on the electrode surface under the 4 V pure-water condition, substrate No. 2 was subjected to thermal treatment and hydrochloric acid immersion (Figure 3a). After heating to 200 °C, some black regions became lighter in color. After heating to 330 °C, the amount of black material decreased markedly, exposing portions of the underlying silver–white electrode surface (Figure 3b). This behavior is consistent with the thermal transformation or decomposition of Ag oxides. If the product contained Ag_2_O, it could decompose into Ag and O_2_ during heating. It should be emphasized that Ag_2_O is generally discussed in terms of its thermal decomposition temperature rather than a melting point, and the decomposition behavior depends on heating rate, sample morphology, and atmosphere. Therefore, the reduction of the black material at 330 °C indicates thermal instability but does not establish that it was a single Ag_2_O phase.

After thermal treatment, the sample was immersed in 0.107 mol/L HCl for 3.5 h. The black particles gradually disappeared, and white flocculent deposits appeared on the electrode surface (Figure 3d). If Ag_2_O was present in the black product, its reaction with hydrochloric acid may be expressed as:

Ag_2_O + 2HCl → 2AgCl↓ + H_2_O (4)

The formation of the white product is consistent with AgCl precipitation. In addition, the solution pH increased from 1 to ~3, indicating acid consumption during immersion. However, the pH change may also have been influenced by residual surface species, dissolved oxygen, and gas exchange with the atmosphere; it cannot therefore be regarded as independent evidence for the presence of Ag_2_O. Taken together, the thermal-treatment and acid-immersion results suggest that the black products may contain Ag_2_O or other oxygen-containing Ag compounds.

CCD observations showed that the black products were mainly distributed on and adjacent to the anode and appeared as discrete particles rather than as a continuous, compact film. This morphology suggests limited surface coverage and barrier capability, such that sustained Ag dissolution was not effectively suppressed. In the presence of a water film and an applied bias, anodic Ag oxidation, transport of mobile Ag-containing species, and cathodic reduction and deposition could therefore continue, ultimately leading to dendritic bridging. These observations should not be interpreted as direct proof that water electrolysis necessarily occurred, because neither current response, gas evolution, nor local pH was measured directly. A more rigorous conclusion is that the applied biases of 3 and 4 V were sufficient to drive redox processes involving the Ag electrodes. Cathodic oxygen reduction and/or water reduction may have occurred, but their respective contributions require further verification through current measurements, gas analysis, or local electrochemical characterization.

### 3.2. Effect of NaCl Concentration on the ECM of Ag

**Figure 4:**
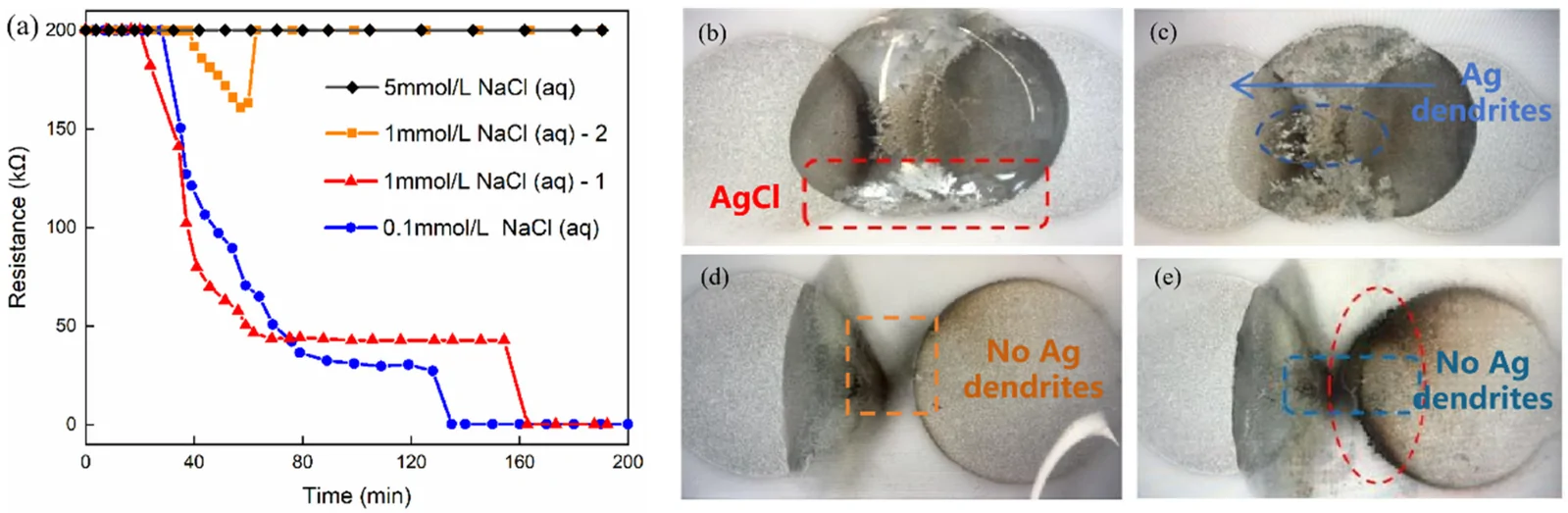
ECM behavior of Ag electrodes at different NaCl concentrations: (**a**) interelectrode resistance as a function of time under different NaCl concentrations; (**b**) CCD image of substrate No. 3 tested in 0.1 mmol/L NaCl, showing dendrites and white deposits; (**c**) CCD image of substrate No. 4 tested in 1 mmol/L NaCl, showing dendritic bridging; (**d**) CCD image of substrate No. 5 tested in 1 mmol/L NaCl, showing no complete dendritic bridge; (**e**) CCD image of substrate No. 6 tested in 5 mmol/L NaCl, showing no dendritic path across the electrode gap.

Salt-spray deposition, saline condensate, and cleaning residues may form Cl^−^-containing liquid films on the surfaces of NTC sensors. Compared with a pure water film, the introduction of NaCl not only increases ionic conductivity but also alters anodic Ag dissolution and the formation pathways of migration products. To evaluate the effect of Cl^−^ concentration, the ECM behavior of Ag was compared in NaCl media with concentrations of 0.1, 1, and 5 mmol/L at room temperature under a 4 V bias (Figure 4a). The role of Cl^−^ in the ECM of Ag cannot be simply classified as either promoting or inhibiting. Under anodic polarization, Ag initially undergoes oxidative dissolution, and the resulting Ag^+^ may react with Cl^−^ to form AgCl precipitates. At higher Cl^−^ activities, AgCl may also form soluble chloroargentate complexes, such as [AgCl_2_]^−^ [14]. Under the present conditions, however, AgCl precipitates, Ag-Cl complexes, and Ag oxide-related products may coexist and undergo dynamic interconversion. Accordingly, the chemical form of migrating Ag species cannot be determined from a single reaction equation, and the notion that low Cl^−^ concentrations promote migration whereas high concentrations inhibit it should not be regarded as universally applicable. The actual effect of Cl^−^ concentration depends on the coupled effects of Ag dissolution rate, continuity of the AgCl deposit layer, liquid-film thickness, electric-field strength, and cathodic reduction kinetics.

For substrate No. 3 tested in 0.1 mmol/L NaCl at room temperature under a 4 V bias, bubbles were observed near both electrodes at the beginning of the test. Because the gas composition was not analyzed, the anodic bubbles cannot be assigned directly to Cl_2_, nor can the cathodic bubbles be identified conclusively as H_2_. At this NaCl concentration, Ag oxidation and water oxidation may both occur at the anode. Whether chlorine evolution is dominant depends on the electrode potential, local Cl^−^ activity, and electrode surface condition. At the cathode, dissolved oxygen reduction and/or water reduction may occur, leading to local alkalization.

After ~20 min of polarization, white flocculent deposits appeared near the cathode, as shown in Figure 4b. Given the presence of Ag and Cl^−^ in the system, the deposits may contain AgCl; however, their phase composition cannot be identified conclusively from color and morphology alone. The deposits may result from the precipitation of anodically generated Ag-containing species after transport to the cathodic region and reaction with Cl^−^. They may also be associated with hydrolysis, deposition, or secondary transformation of Ag species following a local increase in pH. If local Cl^−^ oxidation occurred at the anode, chlorine-containing oxidizing species may also have participated in chemical conversion at the Ag surface. However, since neither gaseous products nor reactive chlorine species in solution were measured, their contributions cannot be distinguished quantitatively.

Despite the presence of white deposits, silver–white dendrites were observed to nucleate at the cathode and grow toward the anode in 0.1 mmol/L NaCl. As shown in Figure 4a, the interelectrode resistance began to decrease after ~35 min and dropped abruptly at ~130 min, indicating that the dendrites eventually formed a continuous conductive path. These results suggest that, at 0.1 mmol/L NaCl, AgCl or other deposition products did not form a sufficiently compact layer to completely suppress sustained Ag dissolution and the transport of mobile Ag-containing species. Active regions remained near the anode, allowing Ag to dissolve, migrate through the liquid film, and undergo reduction and deposition at the cathode.

To qualitatively describe the competition between diffusive transport of Ag-containing species and their capture by AgCl precipitation, a reaction–diffusion characteristic time was introduced. This concept compares the diffusion characteristic time, *τ_diff_*, with the characteristic precipitation time, *τ_AgCl_*. *τ_diff_* can be expressed as:

(5) $$\tau_{\mathrm{diff}}=\;\frac{a{L_{\mathrm{Ag}-\mathrm{Ag}}}^{2}}{D}$$

where *a* is a constant, *D* is the diffusion coefficient of Ag^+^ in aqueous solution (~10^−9^ m^2^ s^−1^), and *L_Ag_*_−*Ag*_ is the interelectrode diffusion distance. The characteristic precipitation time is expressed as:

τ*_AgCl_* = 1/(*k*[*Cl*^−^]) (6)

where *k* is the second-order rate constant. When *τ*_diff_ = *τ*_AgCl_, diffusion and precipitation are balanced. The corresponding reaction–diffusion characteristic length for AgCl precipitation, *L*_AgCl_, can then be expressed as:

(7) $$L_{\mathrm{AgCl}}=\sqrt{D/(a\cdot k\cdot [{\mathrm{Cl}}^{-}])}$$

When *L_AgCl_* < *L_Ag-Ag_*, Ag^+^ is captured by precipitation before reaching the opposite electrode, and passivation tends to dominate. Conversely, when *L_AgCl_* > *L_Ag-Ag_*, Ag^+^ can escape the interfacial region and participate in cathodic reduction at the opposite electrode, favoring migration and dendrite formation. When *L_AgCl_* is comparable to *L_Ag-Ag_*, precipitation and ECM may coexist, and the occurrence of migration-induced short circuit depends on the current and applied voltage.

As shown in Figure 4b, at 0.1 mmol/L NaCl, Ag^+^ could still escape through locally uncovered regions, allowing dendritic growth to continue; under this condition, *L_AgCl_* >> *L_Ag-Ag_*. When the NaCl concentration was increased to 1 mmol/L, substrate No. 4, which exhibited a higher current density, showed a resistance profile similar to that obtained at 0.1 mmol/L NaCl: the resistance began to decrease after ~30 min, and short circuit occurred at approximately 110 min (Figure 4c). In contrast, substrate No. 5, which exhibited a lower current density, showed distinctly different behavior. After an initial, brief decrease in resistance at ~40–60 min, the resistance increased abruptly and then remained high for the rest of the test, indicating complete suppression of short circuiting (Figure 4d).

The coexistence of these two outcomes at the same NaCl concentration suggests that *L_AgCl_* and *L_Ag-Ag_* were comparable under these conditions. Here, the difference in current density should not be interpreted as a change in the intrinsic diffusion coefficient of Ag^+^. Rather, it changes the balance between the Ag dissolution flux and the rate of formation and coverage of AgCl-related deposits. When the anodic dissolution rate was relatively low (substrate No. 5), an AgCl layer could form and cover the anode surface rapidly, suppressing further Ag oxidation and limiting interfacial electron transfer. As the source of mobile Ag species was reduced, dendritic growth could not be sustained, and the resistance recovered and remained high. In contrast, when the anodic dissolution rate was higher (substrate No. 4), the release rate of Ag^+^ exceeded the effective capture rate by precipitation. Some Ag-containing species therefore escaped into the liquid film, were reduced at the cathode, and formed dendrites, resulting in a rapid decrease in resistance and eventual short circuit. Xu et al. [11] similarly reported that the morphology of migration products changed from feather-like to pine-needle-like structures with increasing Cl^−^ concentration, and that AgCl coverage at the anode could retard Ag dissolution and strongly affect whether dendrites formed under critical conditions.

The test of substrate No. 6 in 5 mmol/L NaCl further supports this interpretation. As shown in Figure 4e, the resistance remained high throughout the test, and CCD images showed no dendritic structures between the electrodes. A white AgCl-containing deposit layer and black products were observed at the anode. The black products may be Ag_2_O or other oxygen-containing Ag compounds formed through complex electrochemical reactions under bias; however, their composition was not directly identified. The interelectrode resistance remained approximately 200 kΩ. These results suggest that, at the higher Cl^−^ concentration, *L_AgCl_* << *L_Ag-Ag_*, such that Ag-containing species were more readily captured as AgCl-related deposits than transported to the opposite electrode. Luo et al. [12] reported a similar trend for the ECM of Cu-Ag under NaCl thin-film conditions: cedar-like dendrites formed at NaCl concentrations of ≤6 mmol/L, whereas no dendrites were observed at 100 mmol/L Cl^−^, where precipitation dominated. Overall, in neutral chloride-containing environments, the outcome of ECM is governed by the relative rates of anodic Ag dissolution and AgCl-related deposit formation and coverage. At low Cl^−^ concentrations, dissolution and transport tend to dominate, favoring dendritic short circuiting; at higher Cl^−^ concentrations and lower dissolution rates, precipitation capture and surface coverage become dominant, thereby suppressing migration.

### 3.3. Effect of HCl Concentration on the ECM of Ag

Acidic volatiles or acidic condensates may occur in the vicinity of NTC sensors under conditions such as thermal runaway, material aging, or degradation of encapsulation systems. To investigate the effect of acidic media on the ECM of Ag, HCl solutions adjusted to pH = 5 and pH = 2 were used as test media under a 4 V DC bias at room temperature.

**Figure 5:**
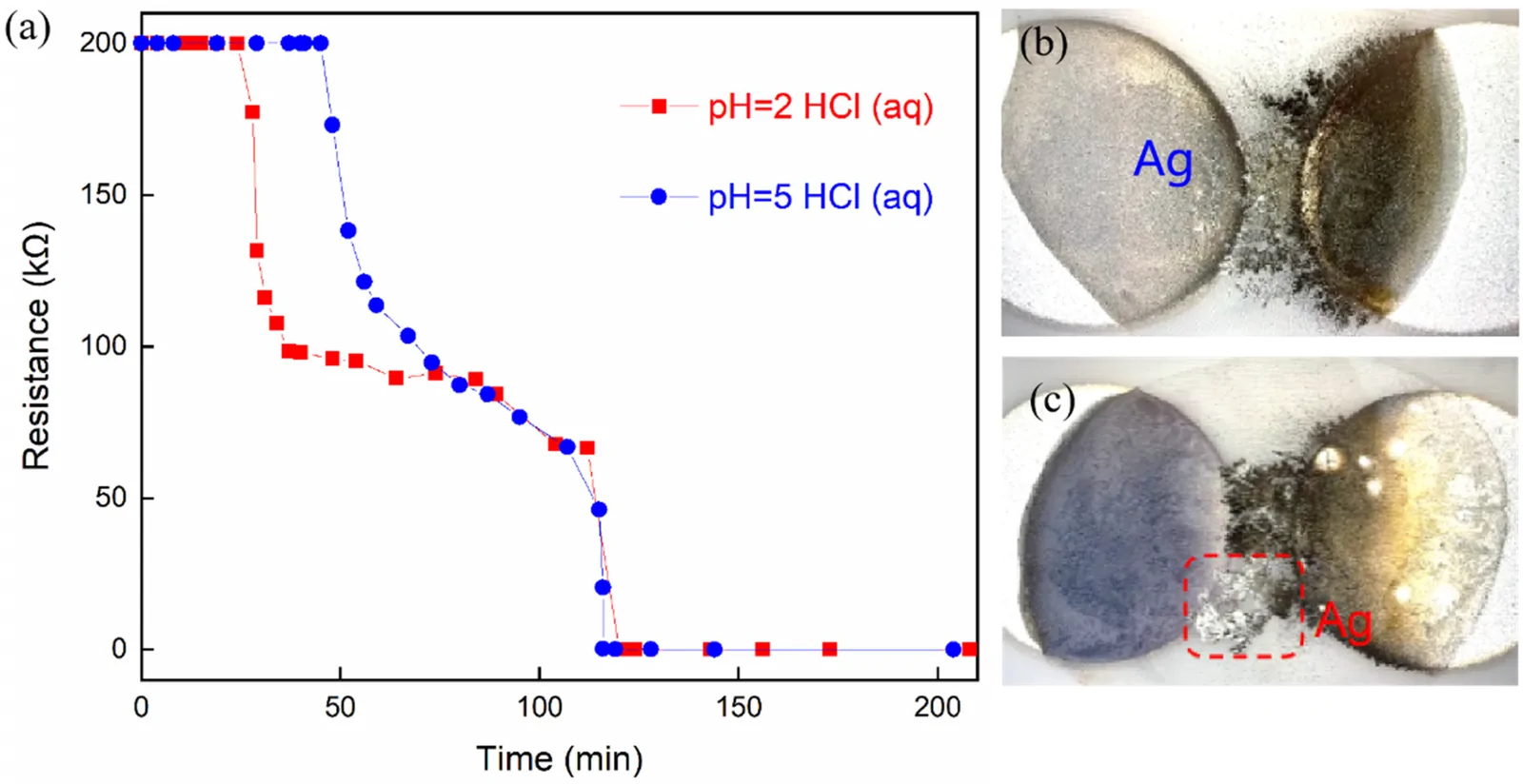
ECM behavior of Ag electrodes in HCl solutions of different acidity: (**a**) interelectrode resistance as a function of time at pH = 5 and pH = 2; (**b**) CCD image of substrate No. 7 after testing in HCl at pH = 5, showing Ag dendritic bridging without an obvious continuous white deposit layer; (**c**) CCD image of substrate No. 8 after testing in HCl at pH = 2, showing Ag dendritic bridging without an obvious continuous white deposit layer.

The resistance profiles of substrates No. 7 (pH = 5) and No. 8 (pH = 2) were broadly similar, as shown in Figure 5a. In both cases, the interelectrode resistance decreased gradually at first and then dropped abruptly, ultimately indicating the formation of a short-circuit path. The short-circuit times were in the range of 110–130 min, comparable to those observed in pure water. CCD observations showed bubbles near the cathode during the initial stage of polarization, with more numerous and larger bubbles at pH = 2. This observation suggests that cathodic reduction became more pronounced with increasing acidity. However, as the gas composition was not analyzed, the bubbles cannot be attributed solely to hydrogen evolution. Cathodic processes may include proton reduction, dissolved oxygen reduction, and water reduction, among which proton reduction has a greater thermodynamic driving force under acidic conditions.

Small amounts of white deposits were observed on the anode during the early stage of the reaction. Their quantity gradually decreased with continued polarization and had largely disappeared after approximately 60 min, exposing the metallic luster of the anode surface. Given the presence of Ag and Cl^−^, the white deposits may be AgCl; however, their identity cannot be confirmed based on color and morphology alone. Notably, the decrease in the white deposits coincided temporally with the rapid reduction in interelectrode resistance and the formation of dendritic bridges, as shown in Figure 5b,c. This result indicates that the surface deposits formed in acidic chloride-containing media did not provide sustained suppression of anodic Ag dissolution. Mobile Ag-containing species could therefore continue to enter the liquid film and undergo reduction and deposition in the cathodic region.

In acidic chloride-containing systems, the stability of an AgCl deposit layer is governed by several factors, including Cl^−^ activity, complexation equilibria of Ag species, interfacial potential, liquid-film renewal, and deposit-layer structure. AgCl can form soluble chloroargentate complexes in the presence of Cl^−^ [15]. It should be noted, however, that the Cl^−^ concentration in HCl at pH = 2 is ~10^−2^ mol/L, which is much lower than that in a 1 mmol/L NaCl solution. Therefore, HCl at pH = 2 should not be regarded as a high-Cl^−^-concentration system, and the disappearance of the white deposits under acidic conditions cannot be explained solely by chloride complexation.

Acidic environments are more likely to influence the formation and persistence of AgCl-related deposits indirectly by modifying the stability of Ag oxides and hydroxides, the nature of cathodic reactions, and local solution chemistry. Compared with neutral or alkaline environments, a higher H^+^ concentration is unfavorable for the stability of Ag oxides, hydroxides, and basic Ag-containing deposits. Consequently, a protective surface layer consisting of AgCl and oxygen-containing Ag species is less likely to develop near the anode. Meanwhile, proton reduction at the cathode may accelerate the cathodic reaction and maintain a higher electron-consumption rate, thereby facilitating sustained anodic oxidation and dissolution of Ag.

In addition, the formation, dissolution, and detachment of surface deposits at the anode may coexist in a dynamic balance under acidic conditions. If the initially formed AgCl layer is thin, poorly adherent, or porous, ions in the solution can still reach the underlying Ag substrate through defects and sustain the anodic reaction. Under the applied electric field and continued ionic transport in the liquid film, the deposit layer may also undergo local dissolution, mechanical detachment, or morphological restructuring, exposing fresh Ag surfaces. Ha et al. [16] reported that the formation rate, coverage, and morphology of AgCl layers strongly affect the corrosion and dissolution behavior of Ag. In conjunction with the present results, it can be inferred that AgCl-related deposits in acidic HCl environments are unlikely to form a stable and continuous barrier over extended periods, and thus cannot effectively limit the release of Ag species into the liquid film.

These results show that both pH = 5 and pH = 2 HCl media favor the sustained ECM of Ag. The transient appearance of white surface deposits under acidic conditions was insufficient to prevent dendrite formation. Anodic Ag dissolution, transport of Ag-containing species, and cathodic reduction and deposition could continue, eventually resulting in dendritic bridging and short-circuit failure between the electrodes.

### 3.4. Effect of Alkaline Carbonate on the ECM of Ag

Battery coolants, cleaning residues, and certain buffer systems may form alkaline liquid films on device surfaces. To simulate such environments, Na_2_CO_3_ solutions adjusted to pH = 9 and pH = 12 were used as test media. The ECM behavior of Ag electrodes was examined at room temperature under a 4 V DC bias.

**Figure 6:**
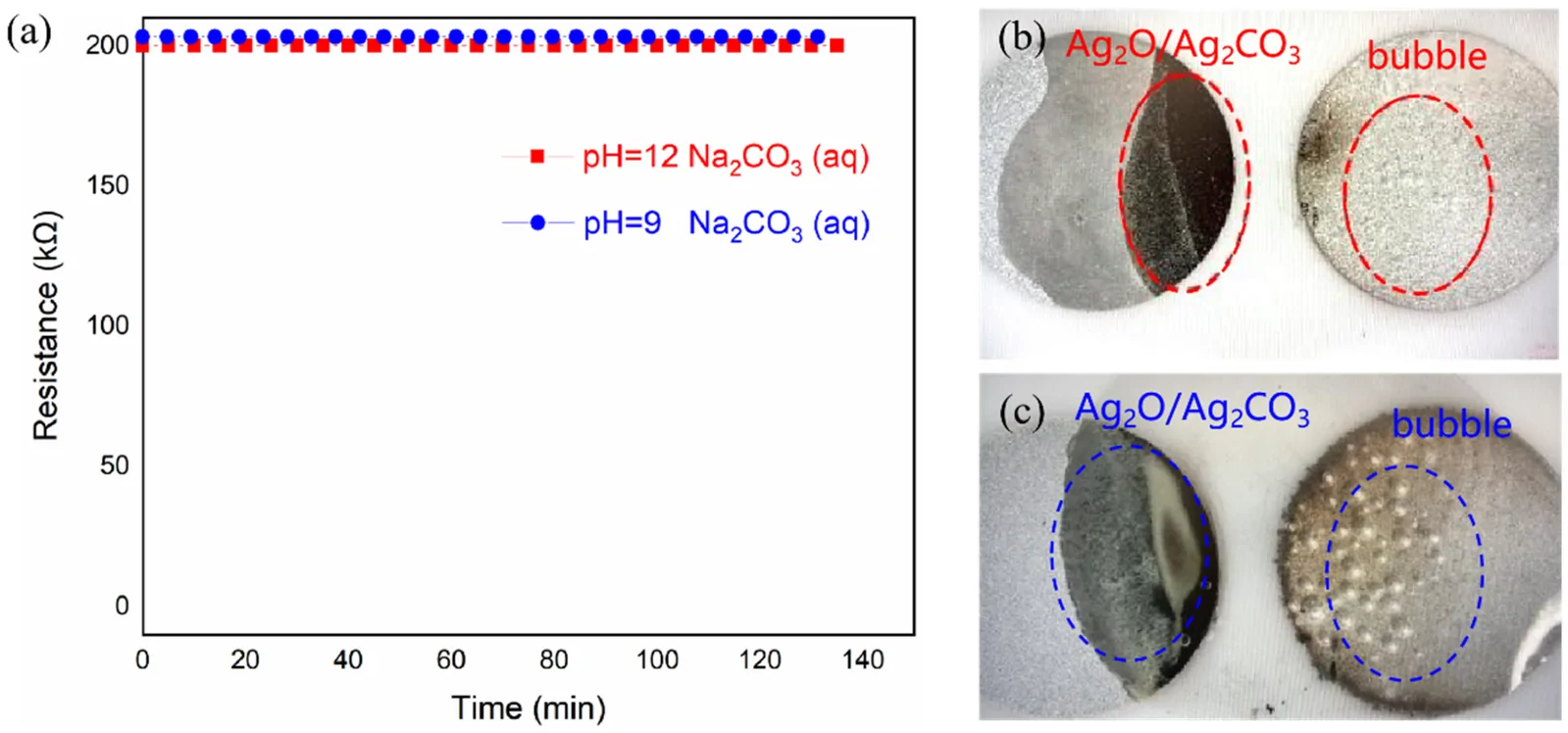
ECM behavior of Ag electrodes in Na_2_CO_3_ solutions at different pH values: (**a**) interelectrode resistance as a function of time at pH = 12 and pH = 9; (**b**) CCD image of substrate No. 9 after testing in Na_2_CO_3_ solution at pH = 12, showing no dendritic bridging and gray–black or gray–white deposits near the anode; (**c**) CCD image of substrate No. 10 after testing in Na_2_CO_3_ solution at pH = 9, showing no dendritic bridging and deposits near the anode.

As shown in Figure 6a, substrates No. 9 (pH = 12) and No. 10 (pH = 9) maintained high interelectrode resistance throughout tests lasting more than 240 min, with neither a sustained decrease nor an abrupt resistance drop. As shown in Figure 6b,c, a small number of bubbles appeared near the cathode. The bubble density was significantly higher at pH = 9 than at pH = 12. This may reflect changes in the cathodic reaction pathway: oxygen reduction may be more important at higher pH, whereas the contribution of water reduction and hydrogen evolution may increase as pH decreases. No silver–white dendrites were observed between the electrodes under either condition.

The evolution of the anode surface provides direct evidence for the ECM suppression. A gray–white surface layer appeared in the anode region after approximately 5–10 min of polarization and gradually thickened with time. Based on the composition of the alkaline carbonate medium and published studies, the deposits may contain Ag_2_O, Ag_2_CO_3_, or a mixture of both. Ibrahim et al. [17] reported that anodic polarization of Ag electrodes in Na_2_CO_3_ solutions can produce surface products associated with Ag oxides and silver carbonate. Possible formation reactions include:

2Ag^+^ + CO_3_^2−^ → Ag_2_CO_3_ (8)

Ag_2_CO_3_ → Ag_2_O + CO_2_ (9)

It should be noted that the latter reaction is more generally associated with thermal decomposition, rather than being an inevitable reaction in an aqueous solution at room temperature. Thus, the surface layer should be described conservatively as an Ag oxide/carbonate-related deposit until direct phase characterization is available.

The formation of this deposit layer is likely central to dendrite suppression. Ag_2_O is a p-type semiconductor with hole-conduction characteristics and is therefore not a perfect electronic insulator. Ag_2_CO_3_ may also exhibit limited electronic conductivity. Consequently, even if the surface layer is relatively compact, it need not completely block charge transfer. This may permit electrochemical reactions to proceed at sufficiently high polarization, while substantially reducing the direct contact between the Ag substrate and the electrolyte.

Gas evolution was observed at the electrode surfaces under the 4 V bias. However, the applied cell voltage cannot be compared directly with the thermodynamic equilibrium potential for oxygen evolution, because the potential distribution between the two electrodes, ohmic drop, and overpotentials were not measured. In alkaline media, anodic Ag oxidation and formation of Ag oxide- or carbonate-related products may compete with water oxidation, while cathodic processes may involve dissolved-oxygen reduction and/or water reduction. Since the composition of the gas was not measured, the anodic bubbles cannot be conclusively assigned to O_2_, and the cathodic bubbles cannot be conclusively assigned to H_2_.

Dendrite formation requires sustained dissolution of Ag^+^ or other mobile Ag-containing species from the anode, followed by their transport through the liquid film and reduction at the cathode. In the present system, the Ag oxide/carbonate-related deposit layer can serve as a physical and chemical barrier that reduces the sustained release of Ag-containing species into the electrolyte. Although the layer may not be perfectly insulating, its formation decreases the availability of mobile Ag species required for cathodic deposition. Consequently, an Ag-ion transport pathway across the electrode gap is not readily established, and dendritic bridging is suppressed.

The stability of such deposits in alkaline carbonate media may be related to the low solubility of Ag-containing oxide and carbonate phases. It should be noted that an Ag-H_2_O Pourbaix diagram alone cannot establish the stability of Ag_2_CO_3_, because carbonate equilibria are not included. Nevertheless, the present results demonstrate that the formation of insoluble Ag-containing deposits in alkaline Ag_2_CO_3_ media effectively suppresses ECM within the tested time scale.

### 3.5. Unified Interpretation Framework for the ECM of Ag

Across all test conditions (Table 2), the formation of Ag dendrites requires two concurrent processes: the sustained release of Ag-containing species from the anode and their transport from the anode to the cathode, followed by cathodic reduction and deposition. Both high-chloride and alkaline environments can suppress this process by limiting the anodic source of mobile Ag species through surface deposition or passivation; however, the nature of the resulting surface layers differs. In chloride-containing media, AgCl-related deposits may hinder Ag dissolution through kinetic surface coverage, whereas in alkaline carbonate media, the surface deposits may contain Ag_2_O, Ag_2_CO_3_, or both. The latter type of deposit is expected to be more stable in alkaline media. In acidic environments, AgCl-related deposits may lose their protective effect because of dissolution, complexation, and structural instability, making dendritic bridging more likely.

At the anodic interface, the fate of dissolved Ag species is governed by competition among three processes: the driving force for anodic dissolution, which promotes the release of Ag species into the liquid film; precipitation and surface capture, which limit their release; and diffusive or electromigrative escape from the interfacial region into the bulk liquid film. Whether passivation is effective therefore depends on whether the formation and coverage of precipitation products can outpace the continued release and escape of Ag species from the anode.

This framework consists of experimental observations. In pure water and 0.1 mmol L^−1^ NaCl, precipitation capture was insufficient to prevent the transport of Ag species, and dendritic growth occurred. At 1 mmol L^−1^ NaCl, dendrites formed under conditions associated with a higher dissolution current, whereas migration was suppressed in the lower-current sample, where AgCl-related surface coverage was more effective. At 5 mmol L^−1^ NaCl, precipitation capture became dominant and no dendritic bridging was observed. Under acidic conditions, the protective effect of AgCl-related deposits was not sustained, allowing continued Ag dissolution and dendrite formation. In alkaline media, the formation of Ag_2_O/Ag_2_CO_3_-related surface deposits substantially limited the release of mobile Ag species, thereby suppressing electrochemical migration.

## 4. Conclusions

This study shows that Ag electrochemical migration requires the presence of a continuous water film and an applied bias. In pure water, increasing the applied voltage accelerated anodic Ag dissolution, the transport of Ag-containing species, and cathodic dendritic deposition, thereby shortening the time to short circuit. The effect of NaCl was concentration dependent. At low NaCl concentrations, mobile Ag species could still traverse the electrode gap and form dendrites. At higher NaCl concentrations, AgCl-related deposits reduced sustained Ag dissolution; however, their inhibitory effect depended on the anodic dissolution flux and the continuity of surface coverage. In acidic HCl media, surface deposits failed to provide a stable protective layer, allowing Ag migration to continue and ultimately causing short-circuit failure. In contrast, insoluble Ag-containing deposits formed in alkaline Na_2_CO_3_ media substantially limited the release of Ag species into the liquid film, and no dendritic bridging was observed during the test period. Overall, the outcome of Ag electrochemical migration is governed by the competition among anodic dissolution, surface deposition/passivation, and interfacial transport of Ag-containing species.

## Figures and Tables

**Table 1:** Test conditions for Ag electrodes under different temperature, humidity, and pH conditions.

Substrate No.	Test Group	Environmental Condition	Voltage	Temperature
1	Low-voltage test	Pure water, drop applied	3 V	80 °C
2	High-voltage test	Pure water, drop applied	4 V	80 °C
3	Low-concentration NaCl	0.1 mmol/L NaCl, 0.001 A	4 V	Room temperature
4	High-concentration NaCl	1 mmol/L NaCl, 0.1 A	4 V	Room temperature
5	High-concentration NaCl	1 mmol/L NaCl, 0.001 A	4 V	Room temperature
6	High-concentration NaCl	5 mmol/L NaCl, 0.001 A	4 V	Room temperature
7	Acidic environment	HCl-adjusted to pH = 5	4 V	Room temperature
8	Acidic environment	HCl-adjusted to pH = 2	4 V	Room temperature
9	Alkaline environment	Na_2_CO_3_-adjusted to pH = 9	4 V	Room temperature
10	Alkaline environment	Na_2_CO_3_-adjusted to pH = 12	4 V	Room temperature
11	Control	No water drop applied	3 V	80 °C

**Table 2:** Test results for Ag electrodes under different temperature, humidity, and pH conditions.

Substrate No.	Environmental Condition	Voltage	Temperature	Result
1	Pure water, drop applied	3 V	80 °C	Short Circuit
2	Pure water, drop applied	4 V	80 °C	Short Circuit
3	0.1 mmol/L NaCl, 0.001 A	4 V	Room temperature	Short Circuit
4	1 mmol/L NaCl, 0.1 A	4 V	Room temperature	Short Circuit
5	1 mmol/L NaCl, 0.001 A	4 V	Room temperature	open circuit
6	5 mmol/L NaCl, 0.001 A	4 V	Room temperature	open circuit
7	HCl-adjusted to pH = 5	4 V	Room temperature	Short Circuit
8	HCl-adjusted to pH = 2	4 V	Room temperature	Short Circuit
9	Na_2_CO_3_-adjusted to pH = 12	4 V	Room temperature	open circuit
10	Na_2_CO_3_-adjusted to pH = 12	4 V	Room temperature	open circuit
11	No water drop applied	3 V	80 °C	open circuit

## Data Availability

The original contributions presented in this study are included in the article. Further inquiries can be directed to the corresponding authors.
